# Intraventricular hemorrhage in reversible cerebral vasoconstriction syndrome

**DOI:** 10.1007/s00415-014-7499-0

**Published:** 2014-09-21

**Authors:** Duncan Wilson, Charles R. Marshall, Thomas Solbach, Laurence Watkins, David J. Werring

**Affiliations:** 1 Stroke Research Group, Department of Brain Repair and Rehabilitation, UCL Institute of Neurology, London, UK; 2 The National Hospital for Neurology and Neurosurgery, Queen Square, Box 6, London, WC1N 3BG UK; 3 Lysholm Department of Neuroradiology, UCL Institute of Neurology, London, UK; 4 Victor Horsley Department of Neurosurgery, National Hospital for Neurology and Neurosurgery, London, UK

Dear Sirs,

A 47-year-old man with no known past medical history presented with a sudden occipital headache, nausea and vomiting. He had been using regular intranasal oxymetazoline for coryzal symptoms during the previous few days. Examination demonstrated left facial weakness but no other neurological abnormality.

**Fig. 1:**
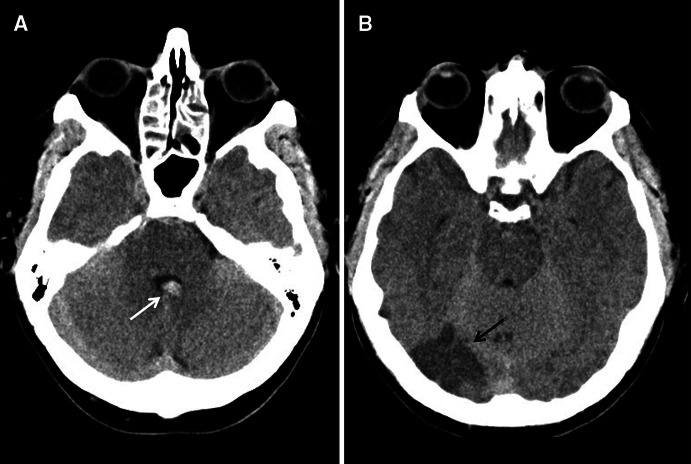
Axial CT scans showing acute intraventricular hemorrhage (**a**, *white arrow*), and right posterior cerebral artery territory infarction (**b**, *black arrow*)

**Fig. 2:**
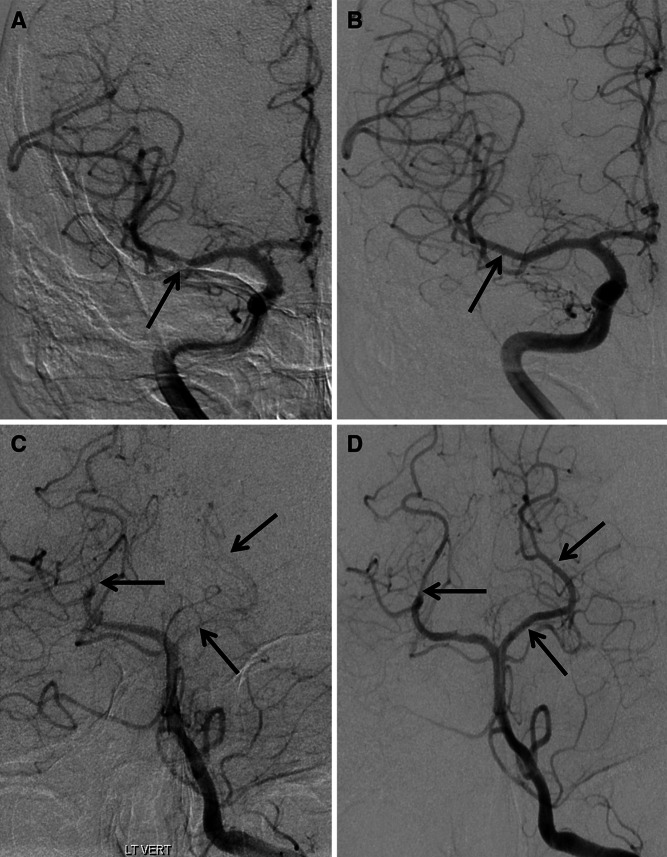
Digital subtraction angiogram. Focal stenosis of right middle cerebral artery (**a**) with resolution after 3 months (**b**). Segmental smooth caliber reduction of both posterior cerebral arteries (**c**) with resolution at 3 months (**d**)

**Fig. 3:**
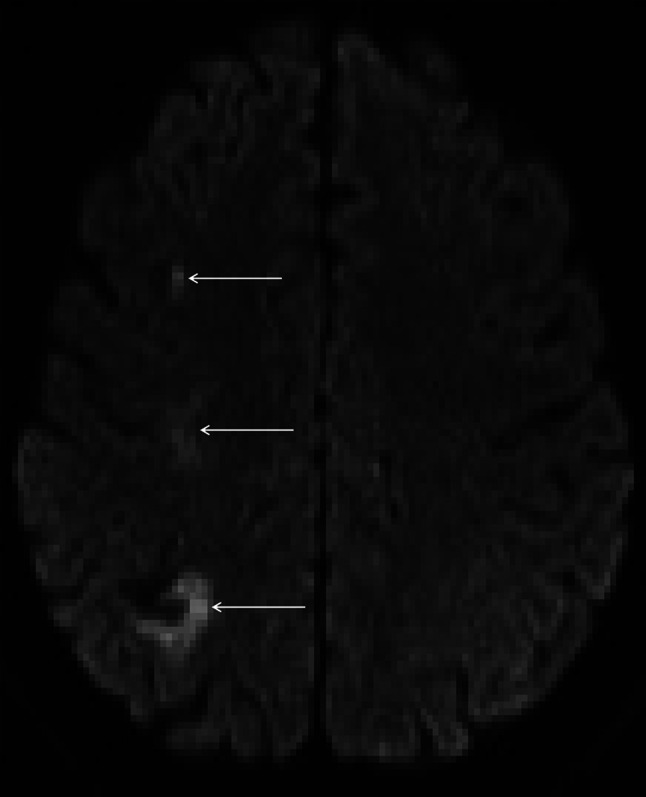
Axial DWI reveals multiple areas of infarction axial DWI reveals ischaemic damage in the right superior parietal lobe, right centrum semiovale and middle frontal sulcus (*white arrows*) suggesting involvement of the internal (subcortical) borderzone territories. In addition there is a small area of gyral haemosiderin staining within the right parietal lobe close to the infarct

Computed tomography showed right posterior cerebral artery infarction and subarachnoid blood in the prepontine cistern and fourth ventricle (Fig. 1). Digital subtraction angiography (DSA) showed irregularities in several intracranial vessels (Figs. 2, 3) but no aneurysm. MRI brain revealed multiple areas of restricted diffusion in the right MCA, PCA and left PCA territories. CSF examination revealed a normal opening pressure, white cell count of 13 cu/mm, red cell count of 7 cu/mm, normal glucose and slightly raised protein of 0.53 g/L. Oligoclonal bands were present but matched in serum.

A presumptive diagnosis of reversible cerebral vasoconstriction syndrome (RCVS) was made. The patient was started on nimodipine 60 mg qds and was advised to avoid further sympathomimetic or nasal decongestant drugs. His neurological examination was normal at discharge, 2 days after his presentation. Repeat DSA 3 months post-admission revealed resolution of the widespread vasoconstriction, confirming a diagnosis of RCVS; the patient remained clinically well, with no further symptoms.

Reversible cerebral vasoconstriction syndrome is typically characterized by recurrent thunderclap headaches, angiographic evidence of vasoconstriction which spontaneously resolves within weeks to months and a normal or near normal CSF examination [1]. The syndrome is rare but likely under-recognized due to lack of systematic angiography in all cases of thunderclap headache [2]. Although many patients have a uniphasic and benign course, cerebral infarction, intracerebral hemorrhage and subarachnoid hemorrhage (usually over the cerebral convexities) are increasingly recognized, and were documented in 34, 20 and 30 %, respectively, in the largest case series to date [3]. Over half the known cases are either associated with the postpartum period or medications with sympathomimetic or serotonergic properties [1]. Although in our case, matched oligoclonal bands were seen in CSF and serum, raising the possibility of vasculitis of the CNS, we found no other supporting evidence of systemic or CNS vasculitis, and the clinical course was typical of RCVS. In contrast to primary angiitis of the CNS (PACNS), a major differential diagnosis, the clinical onset of RCVS is usually abrupt rather than insidious [1]; furthermore whilst headache is a common feature in PACNS, it is almost never of thunderclap type, as is typical of RCVS and seen in our case [4]. The rapid resolution of the clinical syndrome and the angiographic features over days to weeks without corticosteroid treatment is typical of RCVS, and highly unlikely to occur in PACNS [1]. Mild abnormalities in the CSF are not uncommon in RCVS; WCC >10/µL occurred in 8 % of patients in one case series [5] whilst protein levels greater than 60 mg/dL have been found in up to 16 % [3]. We are not aware of any data on the prevalence of oligoclonal bands in RCVS, and such bands may be a non-specific sign of any systemic inflammatory response [6]. Although multiple cerebral infarcts can occur in both conditions, brain imaging in RCVS typically reveals acute infarction in the borderzone territories [1], as seen in our case; by contrast, PACNS typically reveals infarction of different ages involving large and/or branch artery occlusion or a small vessel occlusion pattern [4].

Subarachnoid or intracerebral hemorrhage in RCVS typically occurs over or adjacent to the cerebral convexities in the first few days after the initial headache, often in the setting of normal angiography, and before infarction [5]. These findings have led to the hypothesis that the syndrome initially affects the small superficial leptomeningeal arteries, followed by medium to large territorial arteries [7]. The mechanisms of subarachnoid and intracerebral hemorrhage are not well understood. Vessel rupture or reperfusion injury from vasoconstriction and rapid vasodilation of the leptomeningeal arteries has been postulated [5], whilst the common co-occurrence of hemorrhagic RCVS and posterior reversible vasoconstriction syndrome may implicate endothelial dysfunction in these vessels [5].

To the best of our knowledge, intraventricular hemorrhage has not previously been reported in RCVS. There have been two reports of intraventricular hemorrhage in “postpartum angiopathy” (a condition falling within the disease spectrum of RCVS) [8]; however, unlike the case we describe, both of these cases were in the setting of pregnancy and in association with substantial subarachnoid hemorrhage or parenchymal hemorrhage.

The blood supply to and surrounding the fourth ventricle is largely from small perforators branching from the anterior inferior, posterior inferior and superior cerebellar arteries [9] which are similar in diameter (0.1–1.1 mm) to the leptomeningeal arteries which are hypothesized to be typically affected in hemorrhagic RCVS [10]. Our observation suggests that these small perforators may also be affected by vasoconstriction and reperfusion to cause hemorrhage into the ventricular system. Our findings expand the radiological spectrum of RCVS, which should be considered in the differential diagnosis of intraventricular hemorrhage.
